# Bacterial Communities and Antibiotic Resistance of Potential Pathogens Involved in Food Safety and Public Health in Fish and Water of Lake Karla, Thessaly, Greece

**DOI:** 10.3390/pathogens11121473

**Published:** 2022-12-05

**Authors:** Dimitrios A. Anagnostopoulos, Foteini F. Parlapani, Stamatia Natoudi, Faidra Syropoulou, Maria Kyritsi, Ioannis Vergos, Christos Hadjichristodoulou, Ifigenia Kagalou, Ioannis S. Boziaris

**Affiliations:** 1 Laboratory of Marketing and Technology of Aquatic Products and Foods, Department of Ichthyology and Aquatic Environment, School of Agricultural Sciences, University of Thessaly, Fytokou Street, 38446 Volos, Greece; 2 Laboratory of Hygiene and Epidemiology, Faculty of Medicine, School of Health Sciences, University of Thessaly, Papakyriazi 22 Street, 41222 Larissa, Greece; 3 Management Body of Ecodevelopment Area of Karla, Mavrovouni, Kefalovriso, 37500 Velestino, Greece; 4 Department of Civil Engineering, School of Engineering, Democritus University of Thrace, 67100 Xanthi, Greece

**Keywords:** fish microbiota, Next Generation Sequencing, pathogens, antibiotic resistance, freshwaters, lakes

## Abstract

Bacterial communities, microbial populations, and antibiotic resistance of potential pathogens in the water and fish (*Cyprinus carpio*, flesh and gut) from different areas (A1, A2 and A3—A1 was linked with river water, A2 with cattle activity, and A3 with waters of a spring after heavy rains) of Lake Karla (Thessaly, Central Greece) were investigated. The isolated bacteria were identified using Matrix-assisted laser desorption ionization–time of flight mass spectrometry (MALDI-TOF MS) and were tested for resistance in 21 antibiotics. The microbiota composition of fish flesh was also studied using 16S amplicon-based sequencing *Serratia fonticola* and several species of *Aeromonas* (e.g., *Aeromonas salmonicida*, *Aeromonas bestiarium*, *Aeromonas veronii,* etc.) exhibited the highest abundances in all studied samples, while the microbiota profile between the three studied areas was similar, according to the culture-dependent analysis. Of them, *S. fonticola* was found to be resistant in the majority of the antibiotics for the water and fish (gut and flesh), mainly of the areas A1 and A2. Regarding 16S metabarcoding, the presence of *Serratia* and *Aeromonas* at genus level was confirmed, but they found at very lower abundances than those reported using the culture-dependent analysis. Finally, the TVC and the rest of the studied microbiological parameters were found at acceptable levels (4 log cfu/mL or cfu/g and 2–4 log cfu/mL or cfu/g, extremely low levels of *E. coli*/coliforms) in both water and fish flesh. Based on our findings, the water of Lake Karla would be used for activities such as irrigation, recreation and fishing, however, the development and implementation of a quality management tool for Lake Karla, to ensure environmental hygiene and prevention of zoonosis during the whole year, is imperative.

## 1. Introduction

Pollution affects water quality in lakes around the world. Freshwaters are impacted by incoming chemical (agricultural chemicals, veterinary medicines, or other human-made compounds e.g., plastics) and biological hazards (pathogens), derived by anthropogenic or animal activities of the surrounding areas. Industries and agriculture are the main sources of contamination of the water, sediment, and life in lakes, mostly near urban areas [1,2]. Pathogenic bacteria from livestock systems, cropping systems, pastureland, municipal wastewater sewage systems, etc., can enter lakes, deteriorating water quality, making it sometimes inappropriate for uses such as fishing, aquaculture, irrigation, and recreation. When such pollutants enter the lake environment, it can affect the aquatic biota and fauna. In some cases, such bacteria enter the food value chain and cause illness or death in consumers [3].

Public health is now facing more serious challenges. Pathogenic bacteria containing antibiotic-resistance genes have been increased dramatically in all ecosystems where animals and humans live [3]. The extensive use of antibiotics, mainly in livestock for animal disease management and productivity, has applied a selective pressure to pathogens that live in the environment, resulting in increased antibiotic resistance [4]. Moreover, the continuous contact of such bacteria with antibiotic-contaminated industrial, hospital or agricultural wastes has also facilitated the phenomenon [3,5]. Pathogenic bacteria carrying antimicrobial-resistance genes can be spread anywhere in the terrestrial or aquatic environment [6]. Lakes are among the most significant reservoirs of antibiotic-resistant bacteria, with an additional negative impact on the water quality, food safety and public health. The presence of antibiotic-resistant bacteria in lake water or foods might threaten the efficacy of drugs when these microorganisms colonize in humans.

To reveal the antibiotic-resistant populations, researchers often study the ability of microorganisms to grow on culture media with antibiotic discs on surfaces, or directly detect the genes related with antibiotic resistance. However, before such tests or analyses are carried out, it is essential to differentiate bacterial colonies based on their genome or proteome, in order to know if similar bacteria will or will not present resistance in particular antibiotics. Matrix-assisted laser desorption ionization–time of flight mass spectrometry (MALDI-TOF MS) is a modern cost- and time-effective method [7] used in clinical, environmental and food/seafood microbiology, as well as in other scientific fields [8,9,10,11,12,13]. Moreover, 16S rRNA metabarcoding sequencing has been used to provide a more holistic picture of the microbiota present in seafood [14,15].

Lake Karla, in terms of typology according to the Water Framework Directive 2000/60/EC, is classified as a highly modified water body. Based on the available dataset, the new reservoir is characterized as eutrophic, of poor/bad ecological status, with reduced transparency, high concentration of organic material and nutrients and frequent algal blooms during the dry season. Lakes with high anthropogenic impact and heavy eutrophication always exhibit high potential for antibiotic-resistance genes pollution [16]. In this context, the necessity for providing information to the stakeholders about the potential pathogens and antibiotic-resistant bacteria in the water and fish and where they probably come from, arise. Therefore, the aim of the study was to examine the bacterial communities, potential pathogens and their antibiotic resistance in water and fish from three different sites of Lake Karla; one sampling area linked with river water, one with cattle activity, and one with waters of a spring after heavy rains.

## 2. Materials and Methods

### 2.1. Fish Sampling

Fish nets were placed in three areas of Lake Karla in February 2020 and remained for 24 h in order to collect at least nine individuals of carp (*Cyprinus carpio*) per area. The three areas of sampling were:

*Area 1 (A1).* Entrance of trench 2T to the reservoir (X: 398,702.40, Y: 4,367,499.35). Ditch 2T is the main ditch supplying the reservoir with water from Pinios River during the period October–April.

*Area 2 (A2)* (X: 399,573.67, Y: 4,368,967.49). Point in which there is a strong presence of herds of cattle which graze in riparian areas but also often enter the water, especially during the summer months when the riparian vegetation is reduced to feed on reeds.

*Area 3 (A3)*. Aerani position (X: 401,415.12, Y: 4,369,657.75). Collector Σ6 ends at the point, which supplies the reservoir with the waters of the Kerasiotis spring after heavy rains.

### 2.2. Water Physicochemical Parameters

Conductivity (μS/cm), pH, concentrations of nitrate (NO_3_), nitrite (NO_2_), total phosphorus (TP), total suspended solids (TSS), chloride ions, chemical oxygen demand (COD), 5-day biochemical oxygen demand (BOD5), total ammonia nitrogen, free ammonia and total nitrogen (mg/L) were determined for water from the three sampling points of Lake Karla.

### 2.3. Microbiological Analysis

Twenty-five grams (25 g) of fish tissue or intestines were transferred aseptically to stomacher bags with 90 mL MRD (Maximum Recovery Diluent, 0.1% *w*/*v* peptone, 0.85% *w*/*v* NaCl) and homogenized for 2 min using a stomacher (Bug Mixer, Interscience, London, UK). Volumes (0.1 mL) of 10-fold serial dilutions were spread on the surface of dried media in Petri dishes for enumeration of the following microorganisms: total microbial population as Total Viable Counts (TVC) on TSA (Tryptone Soy Agar), incubated for 48–72 h at 25 °C, *Pseudomonas* spp. on cetrimide–Fucidin–cephaloridine agar (CFC) after incubation at 25 °C for 48 h, *E. coli*/coliforms on *E. coli*/coliform chromogenic medium (HAL008) and *Vibrio* spp. on Thiosulfate–citrate–bile salts–sucrose agar (TCBS), incubated at 37 °C for 24 h. Volumes (1 mL) of 10-fold serial dilutions were used for the pour plate technique for the enumeration of H_2_S-producing bacteria on Iron Agar (IA) by counting only black colonies after incubation at 25 °C for 72 h, Enterobacteriaceae on Violet Red Bile Glucose agar (VRBGA), after incubation at 37 °C for 24 h and Lactic Acid Bacteria (LAB) on De Man Rogosa Sharpe agar (MRS) after incubation at 25 °C for 72 h.

Water analysis was performed using a typical membrane filtration method by passing 100 mL of water through a sterile membrane filter (0.2 µm). Afterwards, the filter was transferred aseptically to the surface of dried media in Petri dishes and incubated at the optimum temperature conditions.

All microbiological media were supplied from LAB M (Lancashire, UK). The results were expressed as mean log cfu g^−1^ ± SD (log colony forming unit per g) of nine replicates for fish flesh and gut and six replicates for the water samples.

### 2.4. Identification Using MALDI-TOF MS

#### 2.4.1. Isolation of Colonies

After the enumeration of counts on the selective culture media (TCBS and *E. coli*/coliforms chromogenic media), more than 50% of the colonies were obtained from each plate (each one containing ~30–300 colonies). Then, each colony was sub-cultured in TSA and incubated at 25 °C for 24–48 h to be used for Matrix-assisted laser desorption ionization–(MALDI) mass spectrometry (MS) analysis.

#### 2.4.2. Sample Preparation for MALDI-TOF MS Analysis

For the identification the MALDI, Microflex LT (Bruker Daltonic GmbH, Bremen, Germany) was used following the on-target-plate protein extraction protocol used as recommended by the manufacturer (Bruker Daltonic) in order to increase spectrum quality. In detail, using a 1 μL sterile inoculating loop a small amount from a freshly grown single colony was directly spotted to a 96-spot steel MALDI target plate and1 µL of 70% formic acid solution (*v*/*v*, 85% AG Moric Acid, Penta, Praha) was carefully applied over each target and left to air-dry. After that, 1 µL of a saturated solution of α-cyano-4-hydroxycinnamomide (HHCA; Bruker Daltonics, Bremen, Germany), was added and allowed to co-crystallize at room temperature.

#### 2.4.3. MALDI-TOF MS Measurements

Spectra were obtained using the linear positive ion mode analysis, with a laser frequency of 60 Hz. The mass range was *m/z* 2000 to 20,000. Parameter settings for Microflex LT were: ion source 1: 20 kV, ion source 2: 18.5 kV, lens: 6 kV, pulsed ion extraction: 100 ns. Spectra were obtained using linear positive mode analysis with laser frequency at 60 Hz. The parameter settings for Microflex LT were: ion source 1: 20 kV, ion source 2: 18.5 kV, lens: 6 kV, pulsed ion extraction: 100 ns. For each spectrum, 240 laser shots were automatically acquired with AutoXecute acquisition control software (FlexControl version 3.4, Bruker Daltonics, Bremen, Germany). The method was externally calibrated using the Bruker Bacterial Test Standard (BTS), a manufactured extract of *Escherichia coli* DH5 alpha spiked with two additional proteins (RNAase A and myoglobin) in order to extend the upper boundary of the mass range covered by BTS. Isolated colonies were identified using MALDI Biotyper Software (version 4.0), and the raw spectra obtained were compared in the mass spectral library (6093 MSPs). The results were categorized according to the manufacturer’s recommended score values. The score value provided by MALDI Biotyper RTC represents the probability that the unknown microorganism is a species in the MALDI Biotyper database. Ιdentification score value 0.000–1.699 represents a not reliable identification, 1.700–1.999 a probable genus identification, 2.000–2.299 a secure genus identification and probable species identification, and finally 2.300–3.000 a highly probable species identification.

### 2.5. Antibiotic Resistance Testing

Each isolated colony was used to inoculate 10 mL TSB (Tryptone Soy Broth), and then incubated at 37 °C for 24 h to ensure that cells were in the stationary phase. A 0.5 McFarland inoculum from each isolate was spread on the surface of a dried Mueller–Hinton agar plate to achieve an initial population of about 10^7^ cfu. Twenty-one (21) different antibiotics in discs, provided by OXOID, in particular, Vancomycin (VA30), Ampicillin (AMP10), Cefoxitin (FOX30), Ceftazidime (CAZ30), Gentamicin (CN10), Ciprofloxacin (CIP5), Tetracycline (TE30), Chloramphenicol (C30), Erythromycin (E15), Oxytetracycline (OT30), Amoxycillin (AML25), Kanamycin (K30), Streptomycin (S10), Nalidixic acid (NA30), Sulphonamides (S3), Clindamycin (DA2), Azithromycin (AZM15), Cephalothin (KF30), Cephazolin (KZ30), Sulphamethoxazole/Trimethoprim (SXT25) and Penicillin G (P10), were placed twice onto the medium surface and incubated at 37 °C for 24 h. Each inhibition zone was measured twice (two plates) in three different diameters. The results were interpreted according to EUCAST Clinical Breakpoint Tables v. 12.0 (valid from 1 January 2022).

### 2.6. 16S Metabarcoding Analysis

#### 2.6.1. Samples Preparation and DNA Extraction

Twenty-five (25) grams of pooled fish flesh (N = 9 fish per area) were transferred aseptically to stomacher bags with 225 mL sterile saline solution (0.85% *w*/*v*, 1:10 dilution) and homogenized for 4 min in a stomacher. Homogenized samples were then transferred to sterile centrifuge tubes and centrifuged (136× *g* for 5 min, 20 °C) to remove any residues. Afterwards, the supernatants were transferred to sterile centrifuge tubes followed by a second centrifugation (2067× *g* for 15 min, 20 °C) and the resulted pellet was diluted in 1 mL of sterile deionized H_2_O.

A total of 200 μL of each diluted pellet was used for bacterial DNA extraction by using the NucleoSpin Tissue kit (Macherey-Nagel GmbH & Co. KG, Düren, Germany), according to the manufacturer’s instructions. Finally, the concentration and quality of the extracted DNA were evaluated on a nanodrop Quawell UV-Vis Spectrophotometer Q5000 (Quawell Technology, Inc., San Jose, CA, USA).

#### 2.6.2. Library Preparation, Sequencing and Bioinformatic Analysis

The metabarcoding analysis was applied according to Syropoulou and et al. [17]. The primers used for the amplification were 27F (AGRGTTTGATCMTGGCTCAG) and the519Rmodbio (GWATTACCGCGGCKGCTG). Amplification conditions were as follows: 95 °C for 5 min, followed by 30 cycles of 95 °C for 30 s, 53 °C for 40 s and 72 °C for 1 min and a final elongation at 72 °C for 10 min. Finally, PCR products were mixed in equal concentration, purified and sequenced on a MiSeq platform following the manufacturer’s protocol.

All bioinformatic analyses (sequences filtering, denoising, chimera checking, taxonomy classification, rarefaction and alpha and beta diversity estimation), were applied using MR DNA ribosomal and functional gene analysis pipeline (www.mrdnalab.com, MR DNA, Shallowater, TX, USA) [17]. Raw sequences were deposited in the National Centre for Biotechnology Information (NCBI), under the Bioproject PRJNA896517.

## 3. Results

### 3.1. Water Physicochemical Parameters

The results of all the physicochemical attributes of water are shown in Table 1. The values of all the parameters from the three areas of the lake did not present statistically significant differences, hence the different lake areas had no effect on the water’s physicochemical properties. Conductivity was measured at 1978, 2458 and 3090 (μS/cm) for areas 1, 2, 3, respectively. The average of the 5-day BOD was 9.23 ± 0.06 (mg/L), while the average of NO_3_^−^ and NO_2_^−^ was 2.78 ± 0.2 mg/L and 0.13 ± 0.01 mg/L, respectively.

### 3.2. Microbiological Analysis

In general, the microbiological profile between the three sampling areas in water, as well as in fish flesh and gut was quite similar, without any noteworthy difference between them (Table 2). However, it is crucial to mention that the microbial load of fish gut was always significantly higher than that of fish flesh and water, the population levels of which were equal. More specifically, the TVC of water and fish flesh showed similar population levels for the three areas, reaching mean values of about 4.02 ± 0.59 log cfu/mL (water) and 4.05 ± 0.70 log cfu/g (fish flesh), while the TVC in the gut reached levels of about 8.88 ± 0.57 log cfu/g. A similar profile was also observed in all the other studied microbiological parameters, since the bacterial counts of fish gut regarding *Pseudomonas* spp. (2.72 ± 0.27, 2.74 ± 0.57 and 8.51 ± 0.55 log cfu/g, for water, flesh and gut, respectively), H_2_S-producing bacteria (2.06 ± 0.08, 3.47 ± 0.63, 8.43 ± 0.57 log cfu/g, for water, flesh and gut, respectively), as well as *Vibrio* spp. (2 ± 0.00, 2.25 ± 0.25, 7.17 ± 0.78 log cfu/g, for water, flesh and gut, respectively), Enterobacteriaceae (2.81 ± 0.33, 2 ± 0.00, 8.57 ± 0.63 log cfu/g, for water, flesh and gut, respectively), coliforms (2.13 ± 0.19, 2 ± 0.00, 7.96 ± 0.67 log cfu/g, for water, flesh and gut, respectively) and *E. coli* (2.11 ± 0.16, 2 ± 0.00, 5.35 ± 0.76 log cfu/g, for water, flesh and gut, respectively), were significantly higher than those of the other two samples (water and fish flesh). Finally, the population levels of lactic acid bacteria were similar in all areas and samples, reaching mean values of about 2.15 ± 0.13, 2.09 ± 0.13 and 2 ± 0.00, for water, flesh and gut, respectively.

### 3.3. Identification of Isolated Bacteria

**Figure 1:**
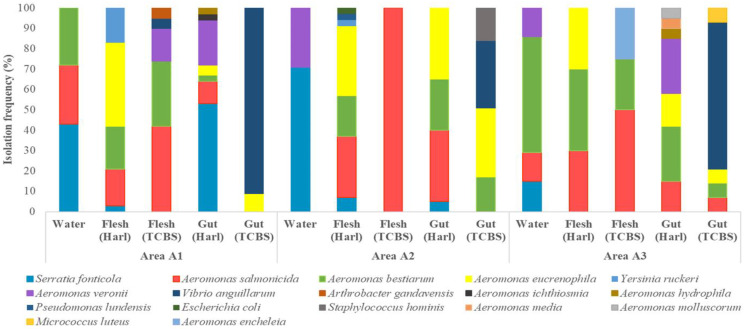
Isolation frequency (%) of the identified isolates through MALDI-TOF MS analysis.

The results of the identification of the isolated bacteria via MALDI-TOF MS analysis were acceptable only when the acquired identification score value was >2000, and they are presented in Figure 1. Overall, *Serratia fonticola* and several species belonging to the genus *Aeromonas* (e.g., *Aeromonas salmonicida*, *Aeromonas bestiarium*, *Aeromonas veronii*, etc.) exhibited by far the highest isolation frequencies in all studied samples, while the microbiota profile between the three studied areas was similar within the majority of the studied parameters (water, flesh and gut). For instance, in water samples the high presence of *S. fonticola* was profound in all three areas (dominant in areas A1 and A2), while the isolation frequency of both *A. salmonicida* (in areas A1 and A3) and *A. veronii* (in areas A2 and A3) is also noteworthy. It is crucial to point out the highest isolation frequency (57%) of *A. bestiarium* in the water of area A3. The latter species was also present in samples from area A1 at relatively high frequency (28%). In addition to water samples, a similar microbial profile was also observed in fish flesh samples from the three studied areas, with *A. eucrenophila*, *A. salmonicida* and *A. bestiarium* being the most representative species, while the presence of *Yersinia suckeri* in flesh from areas A1 and A2, as well as that of *Aeromonas enheltia* in flesh from area A3, should be also highlighted. The same bacteria were included in the main microbial repertoire of the gut samples from all areas, even though *Vibrio auguillarum* exhibited the highest isolation frequency in all gut samples. Finally, it should be underlined that *Micrococcus luteus* has been solely isolated from the gut samples of area A3.

### 3.4. Characterization of Isolates Antibiotic Resistance

The results of antibiotic-resistance testing of all studied isolates (Table 3) indicated a complex profile which could be characterized, in most cases, as isolation origin and microbial species-dependent. However, three main groups can be highlighted based on the isolates’ behavior in each antibiotic.

The first group referred to some antibiotics that have totally hampered the growth of all isolates. Indeed, no bacterial resistance was observed in the presence of vancomycin, clindamycin and penicillin. In this group, amoxylin and ampicillin could be also included, since they inhibited the growth of all isolates, although *V. anguillarum* exhibited resistance to both of them.

The second group includes isolates with partial resistance to some antibiotics, which means that some isolates belonging to the same species were not resistant, while some others exhibited high resistance (no inhibition zone). For instance, this behavior was observed in *S. fonticola*, isolated from water samples (area A1), when exposed to tetracycline (no resistance in 88% of isolates), cephalothin (no resistance in 57% of isolates) and nalidixic acid (no resistance in 88% of isolates).

In the third group, there are antibiotics that did not affect the growth of any of the studied isolates. Those antibiotics are kanamycin, sulphamethoxazole, cefoxitin, gentamicin, ciprofloxacin, streptomycin and azithromycin. In this group, other antibiotics, such as ceftazidime, and sulphonamides, could be also included, although *S. fonticola* and *Y. ruckeri* exhibited partial and/or highly sensitive behavior.

### 3.5. Bacterial Diversity of Fish Flesh

According to 16S rRNA HTS analysis, a total of 40,273 raw reads were obtained (Table S1). Of them, 21,383 were retained after quality filtering, denoising and chimera checking, with an average of 7127 reads per sample (Table S1). The filtered sequences were assigned to a total of 178 observed features (52, 68, 58 for KARL_A1, KARL_A2 and KARL_A3, respectively). The rarefaction to ~2000 sequences indicated a sufficient depth to estimate the bacterial diversity. For instance, the Shannon–Wiener Index curves plot (Figure S1) reached a plateau at approximately 200 sequences in all samples.

Figure S2 illustrates the relative abundances of bacterial diversity at phylum and family level. The results of metataxonomic analysis indicated the dominance of three dominant bacterial phyla (Proteobacteria, Actinobacteria and Cyanobacteria), presented at high abundances among different samples, and another two (Spirochaetes and Firmicutes) which were detected to a lesser extent. More deeply, in samples KARL_A1 and KARL_A3, Proteobacteria was by far the most abundant phylum, even though the presence of Cyanobacteria in sample KARL_A1 is noteworthy. However, it is crucial to mention that zooming in to family level, Pseudomonadaceae followed by Fortieaceae dominated in sample KARL_A1, while Erwiniaceae and Yersiniaceae were by far the most abundant families in sample KARL_A3. On the other hand, Fortieaceae exhibited the highest relative abundance in sample KARL_A2, followed by families belonging to Actinobacteria such as Corynebacteriaceae and Propionibacteriaceae. It should be noted that the presence of Proteobacteria (mainly Aeromonadaceae and Comamonadaceae) was more limited (~10.5%). Finally, both Spirochaetes and Firmicutes were found in traces in all samples, exhibiting relative abundances of no more than 3% in any of the cases.

**Figure 2:**
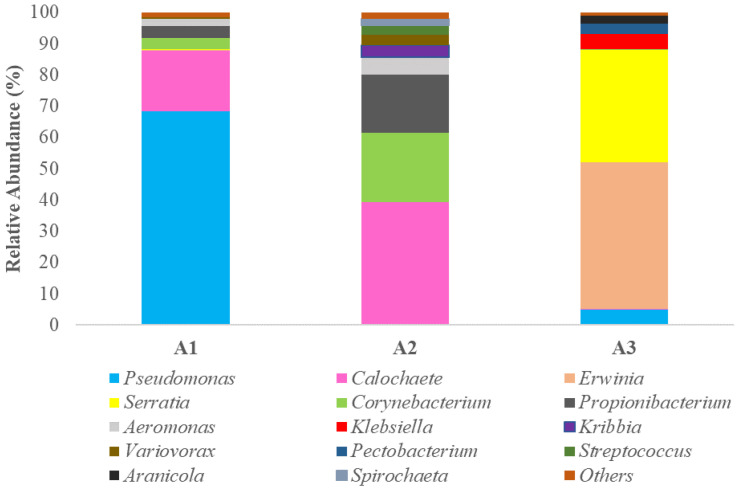
Relative abundance (%) of bacterial genera of the fish flesh from the three different areas (A1, A2 and A3), as revealed by metabarcoding analysis of 16S rRNA gene.

Furthermore, a completely different bacterial profile between samples was observed at genus level (Figure 2). More specifically, in sample KARL_A1, *Pseudomonas* (68.4%) was the most abundant bacterial group, while the presence of *Calochaete* (19.3%) is also noteworthy. However, the latter was the most dominant bacteria genus in sample KARL_A2 (39.2%), while the abundance of *Corynebacterium*, *Aeromonas* and *Propionibacterium* was more limited. In the third sample (KARL_A3) *Erwinia* and *Serratia* co-existed (46.9% and 35.8%, respectively) representing the dominant microbiota, while *Pseudomonas* and *Klebsiella* were found in traces.

## 4. Discussion

In order to prevent the emergence of zoonotic diseases and resolve other environmental issues such as food safety and antimicrobial resistance, nowadays the adoption of a “One Health” approach, that recognizes that the health of humans, domestic and wild animals, plants, and ecosystems are closely related and interacting [18], seems more necessary than ever. Under this perspective, lakes are important sources of water, food, income, and livelihood for millions of people worldwide. However, lakes have been recognized as reservoirs of pathogens, including antibiotic-resistant bacteria, leading to high risks for the ecosystem and human health. Water resources are highly affected by contamination with a wide array of emerging organic compounds (e.g., pharmaceuticals, household chemicals) pathogens and antibiotic-resistant bacteria/genes discharged by Wastewater Treatment Plants (WWTPs) and other points of pollution sources. This phenomenon increases with urbanization and population density as it happens in the Mediterranean area. In the case that a lake is characterized as eutrophic, the possibility of presenting microbial hazards is arisen [16]. Therefore, it is of high priority for us to inform local communities and other stakeholders about the microbiological status of the water and fish from Lake Karla.

Biological pollutants from inland, e.g., animal food processing industries, agriculture, hospitals, pastureland, and municipal waste processing plants, as well as from other sources, e.g., rivers and streams, end up in lakes and contaminate water, threatening aquatic life and public health. Furthermore, water use and re-use are among the cornerstones of the circular economy principles. Among the main concerns are the aquifer’s recharge where the possibility to deteriorate groundwater quality, representing a water body with a very slow turnover time, is very high. This is exactly the case of Lake Karla where the water renewal is very limited especially during the summer dry period. There are very few remediation approaches that can be effectively used to remediate contaminated groundwater. The ecosystem of Lake Karla is strongly linked with various sources of potential contamination, mainly with Pinios River inflow, pastureland/cattle activity, and streams. In general, rivers carry fecal indicators and potential pathogens from human and animal sewages (wild and domestic animals, including food animals) due to the strong anthropic and animal activity in human settlements and megacities, industries, agriculture, etc., across the rivers [19,20,21]. In our case, Pinios River inflow might be a potential means of transporting biological pollutants in Lake Karla since Pinios receives the point outflows of domestic and industrial wastewaters and non-point agricultural return flows [22]. Moreover, cattle activity on the surrounding area of a lake might be responsible for the contamination with serious foodborne enteric pathogens such as *E. coli* O157:H7, *Salmonella*, *Campylobacter*, *Enterococcus*, *Clostridium*, *Bacillus*, etc. [23]. Streams are also causes of transporting such pollutants from soil and road surfaces to rivers and lakes. However, none of the aforementioned bacteria or other serious foodborne or waterborne bacterial pathogens, which are often reported for other lakes in the world, were found in the water or fish (gut nor flesh) from Lake Karla. In parallel, the TVC and the rest of the studied microbiological parameters were found at acceptable levels (4 log cfu/mL or cfu/g and 2–4 log cfu/mL or cfu/g, extremely low levels of *E. coli*/coliforms) both in water and fish flesh, showing that the water of Lake Karla would be used for activities such as irrigation, recreation and fishing. However, this observation was performed in cold months in Greece (average maximum temperature: 13 °C or 55 °F). In summer months (average maximum temperature: 32 °C or 90 °F), fish from all sampling areas of Lake Karla (A1, A2 and A3) were sick (reddish, swollen abdomen and diffuse intestines) and dominated by *Plesiomonas shigelloides*, followed by *A. veronii* and *A. hydrophila* in gut and flesh, equally (data not shown), showing that higher water temperature acting synergistically with low water level and organic pollutants and might increase the abundance of bacterial pathogens responsible for zoonosis. Among them, *Pl. shigelloides* is a fish pathogen [24,25,26,27] as well as a human pathogen, causing various diseases such as gastroenteritis, bacteremia, meningitis, pneumonia, osteomyelitis, sepsis and keratitis, if ingested [28]. *Pl. shigelloides* has exhibited resistance to multiple antibiotics [29,30] as well as *Aeromonas* species [31], raising public health concerns. It is also worth mentioning that human infections occur during summer months and are associated with environmental contamination of freshwater bodies [32], which can explain why only fish caught in July were sick. An improvement of the water quality and improvement of the ecological status at least to the “good” category is essential in order to avoid zoonosis and thus, the development and implementation of a quality management tool for Lake Karla is imperative.

*S. fonticola,* a member of the Enterobacteriaceae family and species belonging to the *Aeromonas* genus, had the highest isolation frequency in all studied samples, while the microbiota profile of the three different areas were similar between the studied parameters (water, flesh and gut). Among these bacteria, *S. fonticola* was found in all three areas (dominant in areas A1 and A2), while *A. salmonicida* and *A. veronii* dominated in A1, A3 and A2, A3 areas, respectively. Although there is little information in the literature regarding *S. fonticola* physiology, it is considered an opportunistic human pathogen primarily described as causing skin and soft tissue infections following trauma [33]. On the other hand, *A. veronii*, *A. hydrophila* and *A. caviae* cause infections such as bacteremia, gastroenteritis and septicemia in immunocompetent and immunocompromised individuals [34]. Moreover, more and more fish diseases have been caused by several *Aeromonas* species, such as *A. caviae* [35], *A. veronii* [36], *A. salmonicida* [37], *A. hydrophila* [38], *A. sobria* [39] and *A. bestiarum* [40]. Among them, *A. hydrophila* had been considered to be the most harmful microorganism for aquatic animals, frequently causing hemorrhagic disease in farmed fish [41,42]. However, more recently, *A. veroni* have increasingly been infecting fish, with many similar symptoms and histological lesions compared to *A. hydrophila*.

The genus of *Serratia*, including *S. fonticola*, features intrinsic and acquired resistance to a variety of antibiotic families. Their resistome includes intrinsic resistance genes that offer a “natural” resistance to β-lactam family antibiotics, to polypeptides and to quinolones, while they lack resistance genes for trimethoprim and sulfonamides. Finally, resistance to aminoglycosides seems to be acquired by some strains [43,44]. In a study conducted by Sandner-Miranda and co-workers [45], all *Serratia* species tested were uniformly naturally resistant to penicillin G, oxacillin, cefazolin, cefuroxime, all tested macrolides, lincosamides, streptogramins, glycopeptides, fusidic acid and rifampicin, and naturally sensitive to several aminoglycosides, piperacillin, piperacillin/tazobactam, carbapenems, some cephalosporins, fluoroquinolones and folate-pathway inhibitors. The same study demonstrated that most of the resistomes of clinical and environmental bacteria of the genus *Serratia* share approximately similar number of antimicrobial-resistance genes (ARGs) and their differences result from the content of the horizontally transferred genes.

Although *S. fonticola* has been reported to be susceptible to extended-spectrum cephalosporins, fluoroquinolone and carbapenem, some strains isolated from clinical specimens have been found to be resistant to all groups of antibiotics [46]. In our study, the results of the susceptibility testing of the strains of *S. fonticola* from the areas A1 and A2, linked with river water and with cattle activity, respectively, were in accordance with the resistance profile as described above, displaying resistance in most agents examined. Furthermore, the strains isolated in water, fish flesh and fish gut displayed the same antibiotic-resistance profile, denoting the potential horizontal transfer of ARGs.

*Aeromonas* spp. are ubiquitous in an aquatic environment and they can acquire, exchange and act as potential reservoirs of ARGs. That is why many researchers have used the microorganisms as indicators for monitoring AMR in these environments. *Aeromonas* isolates are usually intrinsically resistant to ampicillin, and Zdanowicz et al., [31] found *Aeromonas* to be resistant to amoxicillin, ampicillin, clindamycin and penicillin. *Aeromonas* spp. can also serve as contributors to horizontal gene transfer, both as donors and recipients, because of their abundance in water and antibiotic-rich environments (i.e., humans, fish farms, and agriculture). In general, *Aeromonas* spp. have shown different levels of resistance to antibiotic agents depending on the contamination of their environment from human and/or agricultural pollutants. In our study, the strains of *Aeromonas* did not display resistance to all agents tested, a fact that deserves further attention in the near future, since it was expected by the nature of the microorganism to display remarkable resistance, according to the literature. *V. anguillarum* was also found in the gut of all studied samples, so we should raise the awareness of all stakeholders, including citizens, to ensure the quality of the lake water is at levels that prevent the high presence of this bacterium. *V. anguillarum* is the main agent of vibriosis, a major hemorrhagic septicemic disease affecting various seafood worldwide, leading to noteworthy economic losses for the producers [47]. The ability of this bacterium to cause fish disease depends on the presence of virulence factors, as well as on a tight control of their expression [48]. The latter requires more deeply scientific attention to control the outbreaks of fish disease caused by the activity of this bacterium, to ensure fish safety and prevent economic losses.

It is also crucial to mention that the study of gene expression involved in antibiotic resistance of the isolated bacteria, via the use of modernized molecular methods such as qPCR and/or RNA-Seq, constitutes an interesting scientific field that deserves considerable attention in the near future.

Metabarcoding analysis was applied only in fish flesh, since this is what is consumed and reaches the consumer’s plate as food. Results indicated a noteworthy different bacterial profile between the samples from the three different areas, indicating that agricultural/industrial activities near each area’s watershed greatly affect the quality and microbial profile of the water and consequently, the microbial repertoire, the quality and the safety of fish flesh. In particular, *Pseudomonas* was the most abundant bacterial group in sample KARL_A1, while *Calochaete* was present at a lesser percentage. Nevertheless, the latter was the most dominant bacterial group in sample KARL_A2, followed by *Corynebacterium*, *Aeromonas* and *Propionibacterium*. In the KARL_A3 sample, *Erwinia* and *Serratia* co-existed as the dominant microbiota, while *Pseudomonas* and *Klebsiella* were found in lower abundances. Several species belonging to *Pseudomonas* genus are usually found in fish flesh as a major part of its natural environmental-originated microbiota [15]. This genus has been repeatedly found in fresh fish from the Hellenic basin [49,50,51,52], in parallel one with the major and well-known Specific Spoilage Organisms (SSOs) that cause the rapid spoilage of fish [53,54]. It has also been described as one of the most common bacterial infectious agents of fish, since the high presence of *Pseudomonas* spp. is closely linked to stress-related diseases in freshwater fish [55,56]. Regarding *Calochaete*, which was found at noteworthy abundance in sample KARL_A2, to our knowledge, this is the first study reporting this cyanobacterium as a main part of fish microbiota. The literature is limited regarding this microorganism, of which its presence and role in fish flesh deserve further attention in the near future.

Several *Aeromonas* species can cause infection after host injury or stress response [41,42,57], in parallel causing various infections in humans [58]. Furthermore, several fish disease outbreaks have been caused by this bacterium [35,37]. The aforementioned indicated that the high presence of this bacterium in fish flesh deserves major attention, since it raises health and safety issues for consumers. Regarding *Corynebacterium*, according to [59], this genus includes several species with or without pathogenicity. For instance, the pathogens including species such as *C. accolens* [60] and *C. bovis* [61] have been reported to cause mastitis. However, other species such as *C. glutamicum*, *C. efficiens* [62] and *C. glycinophilum* have been found to be not only non-pathogenic but stimulate food and amino acid production. In this sense, further work implementing molecular methods to reach species- and/or strain-level identification is needed in order to reveal which *Corynebacterium* species are present in fish from area A2, and thus estimate the risk level of consuming those fish. Referring to *Propionibacterium*, this genus is another environment-originated bacterium that is widespread in aquatic habitats, and consequently in fish flesh [15]. There is evidence that some species, such as *P. acnes,* are known primarily as skin commensals and are considered as opportunistic pathogens, causing invasive infections such as implant-associated infections [63]. A study focusing on plant protein digestion in salmonids demonstrated that a group of fish fed soy protein concentrate had intestinal disorders at high seawater temperatures and coincidently experienced increased bacterial diversity, including bacteria such as *Propionobacterium*, the high presence of which is not normally associated with fresh fish [64]. Finally, *Serratia* spp., which was found at high abundance in the sample from area A3 (KARL_A3), is a common bacterial genus presented in soil and freshwater [65]. However, the emergence of multidrug-resistant *Serratia* has been alarming not only to the medical field but also to the fish production and aquaculture sector [66]. Similar to this, *Erwinia,* which was also recorded at high presence in sample KARL_A3, has been reported as a potential pathogen of some fish species, since it has been closely linked to, e.g., salmon disease [67]. This bacterium is also related to fish spoilage via its ability to produce unpleasant odors and high amounts of trimethylamine (TMA) [68]. 

The spread of pathogens and of antibiotic-resistant bacteria (ARB) and antibiotic-resistance genes (ARGs) is considered one of the most emerging threats to public health. To tackle this challenge in a comprehensive way and to develop multidisciplinary and practical solutions for the provision of healthy aquatic environments, a monitoring strategy based on a risk assessment approach should be implemented in compliance with the already existing water policy legislation. Yet, modelling tools leading to Decision Support Systems (DSS) would allow policy makers to estimate the performance of the plan and provide regulations. Furthermore, a holistic catchment approach for a better understanding of the ecological and human and animal health effects is necessary.

The pillar of water protection in the European Union (EU) is Directive 2000/60/EC, known as the EU Water Framework Directive (WFD), along with “sister” directives (Flood, Bathing, Wastewater Directives), while the World Health Organization WHO (WHO, 2017) recommended developing minimum quality requirements for the safe use of reclaimed water for agricultural irrigation and aquifer recharge as a risk management framework. On a global scale, the United Nations (UN) Sustainable Development Goals (SDGs) have set out a sustainable blueprint for the international community to improve human health, ensure provision of safe water for all and safeguard both marine and freshwater ecosystems by 2030.

## Figures and Tables

**Table 1:** Physicochemical parameters of the water of Lake Karla in February 2020.

Area	Conductivity (μS/cm)	pH (20 °C)	NO_3_^−^ (mg/L)	NO_2_^−^ (mg/L)	TP (mg/L)	TSS (mg/L)	Cl^−^ (mg/L)	COD (mg/L)	BOD5 (mg/L)	Total NH_3_^+^ (mg/L)	Free NH_3_^+^ (mg/L)	TN (mg/L)
A1	1978	8.1	2.60	0.14	0.10	294	1240	30	9.3	0.12	0.09	3.60
A2	2458	8.2	2.75	0.14	0.10	234	1485	28	9.2	0.11	0.08	3.75
A3	3090	8.3	3.00	0.12	0.10	218	1995	28	9.2	0.11	0.07	3.80

**Table 2:** Microbiological profile (TVC, *Pseudomonas* spp., H_2_S-producing bacteria, Enterobacteriaceae, Coliforms, *E. coli*, Lactic acid bacteria, and *Vibrio* spp.) of water, fish flesh and fish gut samples from the 3 different areas (A1, A2 and A3). Each data point shows the mean and ± st. dev. of 6 and 9 replicates for the water and fish (tissue, gut), respectively.

Microorganism	Area	Water	Flesh	Gut
TVC	A1	3.79 ± 0.68	4.13 ± 0.69	8.83 ± 0.60
	A2	3.94 ± 0.65	3.31 ± 0.72	8.99 ± 0.66
	A3	4.34 ± 0.43	4.71 ± 0.70	8.81 ± 0.46
*Pseudomonas* spp.	A1	2.87 ± 0.18	3.07 ± 0.72	8.64 ± 0.79
	A2	2.76 ± 0.26	2.12 ± 0.25	8.57 ± 0.41
	A3	2.54 ± 0.37	3.05 ± 0.75	8.33 ± 0.47
H_2_S-producing bacteria	A1	2.05 ± 0.07	3.52 ± 0.63	8.68 ± 0.66
	A2	2.14 ± 0.18	2.89 ± 0.58	8.35 ± 0.55
	A3	2.00 ± 0.00	4.00 ± 0.68	8.25 ± 0.50
Enterobacteriaceae	A1	2.71 ± 0.53	2.00 ± 0.00	8.55 ± 0.53
	A2	2.97 ± 0.19	2.00 ± 0.00	9.00 ± 0.51
	A3	2.74 ± 0.26	2.00 ± 0.00	8.18 ± 0.83
*E.coli*/coliforms	A1	2.00 ± 0.00	2.00 ± 0.00	8.51 ± 0.78
	A2	2.13 ± 0.18	2.00 ± 0.00	8.94 ± 0.49
	A3	2.27 ± 0.39	2.00 ± 0.00	6.42 ± 0.73
*E. coli*	A1	2.00 ± 0.00	2.00 ± 0.00	5.53 ± 0.50
	A2	2.05 ± 0.08	2.00 ± 0.00	5.60 ± 0.99
	A3	2.28 ± 0.39	2.00 ± 0.00	4.92 ± 0.78
Lactic acid bacteria	A1	2.00 ± 0.00	2.26 ± 0.40	2.00 ± 0.00
	A2	2.45 ± 0.40	2.00 ± 0.00	2.00 ± 0.00
	A3	2.00 ± 0.00	2.00 ± 0.00	2.00 ± 0.00
*Vibrio* spp.	A1	2.00 ± 0.00	2.74 ± 0.76	7.42 ± 0.71
	A2	2.00 ± 0.00	2.00 ± 0.00	7.16 ± 0.77
	A3	2.00 ± 0.00	2.00 ± 0.00	6.92 ± 0.86

**Table 3:** Antibiotic-resistant bacteria in water (W), fish flesh (F) and fish gut (G) from the three different sampling areas (A1, A2 and A3) of Lake Karla. * For bacterial species presented both resistant and susceptible isolates.

A1	*Serratia fonticola*	*Yersinia ruckeri*	*Vibrio anguillarum*	*Aeromonas salmonicida*	*Aeromonas bestiarum*	*Aeromonas eucrenophila*	*Aeromonas veronii*	*Aeromonas ichthiosmia*	*Aeromonas hydrophila*	*Aeromonas encheleia*	*Aeromonas media*
CAZ30	W, F, G	F	NR	NR	NR	NR	NR	NR	NR	NR	NR
DA2	-	-	F, G	-	-	-	-	-	-	-	-
K30	NR	NR	NR	-	-	-	-	-	-	-	-
E15	-	-	NR	-	-	-	-	-	-	-	-
SXT25	NR	NR	NR	NR	NR	NR	NR	NR	NR	NR	NR
VA30	-	-	F, G	-	-	-	-	-	-	-	-
FOX30	NR	NR	NR	-	-	-	-	-	-	-	-
OT30	-	-	NR	-	-	-	-	-	-	-	-
CN10	NR	NR	NR	-	-	-	-	-	-	-	-
CIP5	NR	NR	NR	NR	NR	NR	NR	NR	NR	NR	NR
AML25	W, F, G	F	NR	-	-	-	-	-	-	-	-
TE30	-	-	NR	-	-	-	-	-	-	-	-
KF30	W *, G *	F	NR	-	-	-	-	-	-	-	-
KZ30	W, F, G *	F	NR	-	-	-	-	-	-	-	-
NA30	W *, F, G *	F*	NR	-	-	-	-	-	-	-	-
S10	NR	F*	NR	-	-	-	-	-	-	-	-
P10	-	-	F, G	-	-	-	-	-	-	-	-
AZM15	-	-	NR	-	-	-	-	-	-	-	-
C30	NR	NR	NR	-	-	-	-	-	-	-	-
AMP10	W, F, G	F	F	-	-	-	-	-	-	-	-
S3	G *	F	NR	-	-	-	-	-	-	-	-
**A2**	***Serratia fonticola***	***Yersinia ruckeri***	***Vibrio anguillarum***	***Aeromonas salmonicida***	***Aeromonas bestiarum***	***Aeromonas eucrenophila***	***Aeromonas veronii***	***Aeromonas ichthiosmia***	***Aeromonas hydrophila***	***Aeromonas encheleia***	***Aeromonas media***
CAZ30	W, F, G	F	NR	NR	NR	NR	NR	NR	NR	NR	NR
DA2	-	-	G	-	-	-	-	-	-	-	-
K30	NR	NR	NR	-	-	-	-	-	-	-	-
E15	-	-	NR	-	-	-	-	-	-	-	-
SXT25	NR	NR	NR	NR	NR	NR	NR	NR	NR	NR	NR
VA30	-	-	G	-	-	-	-	-	-	-	-
FOX30	NR	NR	NR	-	-	-	-	-	-	-	-
OT30	-	-	NR	-	-	-	-	-	-	-	-
CN10	NR	NR	NR	-	-	-	-	-	-	-	-
CIP5	NR	NR	NR	NR	NR	NR	NR	NR	NR	NR	NR
AML25	W, F, G	F	NR	-	-	-	-	-	-	-	-
TE30	-	-	NR	-	-	-	-	-	-	-	-
KF30	F,	F	NR	-	-	-	-	-	-	-	-
KZ30	W, F, G	F	NR	-	-	-	-	-	-	-	-
NA30	W, F, G *	F	NR	-	-	-	-	-	-	-	-
S10	NR	NR	NR	-	-	-	-	-	-	-	-
P10	-	-	G	-	-	-	-	-	-	-	-
AZM15	-	-	NR	-	-	-	-	-	-	-	-
C30	NR	NR	NR	-	-	-	-	-	-	-	-
AMP10	W, F, G	F	NR	-	-	-	-	-	-	-	-
S3	G *	F	NR	-	-	-	-	-	-	-	-
**A3**	***Serratia fonticola***	***Yersinia ruckeri***	***Vibrio anguillarum***	***Aeromonas salmonicida***	***Aeromonas bestiarum***	***Aeromonas eucrenophila***	***Aeromonas veronii***	***Aeromonas molluscorum***	***Aeromonas hydrophila***	***Aeromonas encheleia***	***Aeromonas media***
CAZ30	NR	NR	NR	NR	NR	NR	NR	NR	NR	NR	NR
DA2	-	-	G	-	-	-	-	-	-	-	-
K30	NR	NR	NR	-	-	-	-	-	-	-	-
E15	-	-	NR	-	-	-	-	-	-	-	-
SXT25	NR	NR	NR	NR	NR	NR	NR	NR	NR	NR	NR
VA30	-	-	G	-	-	-	-	-	-	-	-
FOX30	NR	NR	NR	-	-	-	-	-	-	-	-
OT30	-	-	NR	-	-	-	-	-	-	-	-
CN10	NR	NR	NR	-	-	-	-	-	-	-	-
CIP5	NR	NR	NR	NR	NR	NR	NR	NR	NR	NR	NR
AML25	W	NR	NR	-	-	-	-	-	-	-	-
TE30	-	-	NR	-	-	-	-	-	-	-	-
KF30	NR	NR	NR	-	-	-	-	-	-	-	-
KZ30	W	NR	NR	-	-	-	-	-	-	-	-
NA30	W	NR	NR	-	-	-	-	-	-	-	-
S10	NR	NR	NR	-	-	-	-	-	-	-	-
P10	-	-	G	-	-	-	-	-	-	-	-
AZM15	-	-	NR	-	-	-	-	-	-	-	-
C30	NR	NR	NR	-	-	-	-	-	-	-	-
AMP10	W	NR	NR	-	-	-	-	-	-	-	-
S3	NR	NR	NR	-	-	-	-	-	-	-	-

NR: No resistance. Symbol (-): The antibiotic is not used for this bacterium according to EUCAST Clinical Breakpoint Tables v. 12.0, valid from 1 January 2022. Vancomycin (VA30), Ampicillin (AMP10), Cefoxitin (FOX30), Ceftazidime (CAZ30), Gentamicin (CN10), Ciprofloxacin (CIP5), Tetracycline (TE30), Chloramphenicol (C30), Erythromycin (E15), Oxytetracycline (OT30), Amoxycillin (AML25), Kanamycin (K30), Streptomycin (S10), Nalidixic acid (NA30), Sulphonamides (S3), Clindamycin (DA2), Azithromycin (AZM15), Cephalothin (KF30), Cephazolin (KZ30), Sulphamethoxazole/Trimethoprim (SXT25) and Penicillin G (P10).

## Data Availability

The datasets generated for this study are available on request to the corresponding author.

## Supplementary Materials

The following supporting information can be downloaded at: https://www.mdpi.com/article/10.3390/pathogens11121473/s1, Table S1: Number of raw and filtered reads and alpha diversity indices of evaluated samples; Figure S1: Shannon–Wiener rarefaction curves of the of the fish flesh from the three different areas (A1, A2 and A3) revealed by 16S rRNA metabarcoding analysis through 10 sampling depths; Figure S2. Relative abundance (%) of bacterial phyla (upper) and families (down) of the fish flesh from the three different areas (A1, A2 and A3), as revealed by metabarcoding analysis of 16S rRNA gene.
