# Supplementary material for: Bacterial Communities and Antibiotic Resistance of Potential Pathogens Involved in Food Safety and Public Health in Fish and Water of Lake Karla, Thessaly, Greece
Source: Pathogens. 2022 Dec 5;11(12):1473. doi: 10.3390/pathogens11121473 (PMC9785323; doi:10.3390/pathogens11121473)
Supplement: Supplementary file 1 [file pathogens-11-01473-s001.zip › pathogens-2048251-supplementary.pdf]

Table S1. Number of raw and filtered reads and alpha diversity indices of evaluated samples.

Sample	Number of reads	Filtered sequences	Observed Features	Shannon
C_KARL_A1	6,802	2,989	52	4.29
C_KARL_A2	4,745	4,185	68	5.23
C_KARL_A3	28,726	14,209	58	3.49

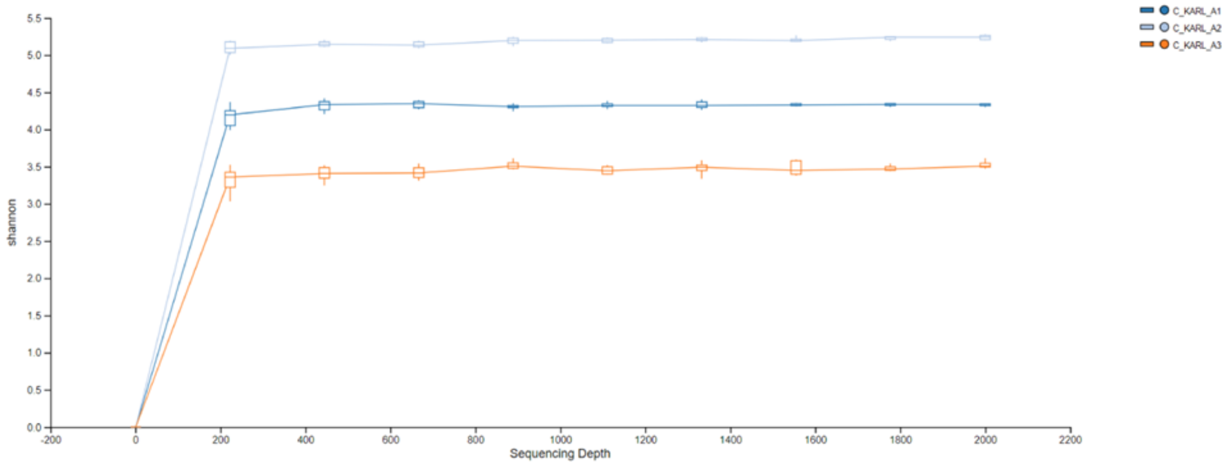

Figure S1. Shannon-Wiener rarefaction curves of the of the fish flesh from the three different areas (A1, A2 and A3) revealed by 16S rRNA metabarcoding analysis through 10 sampling depths.

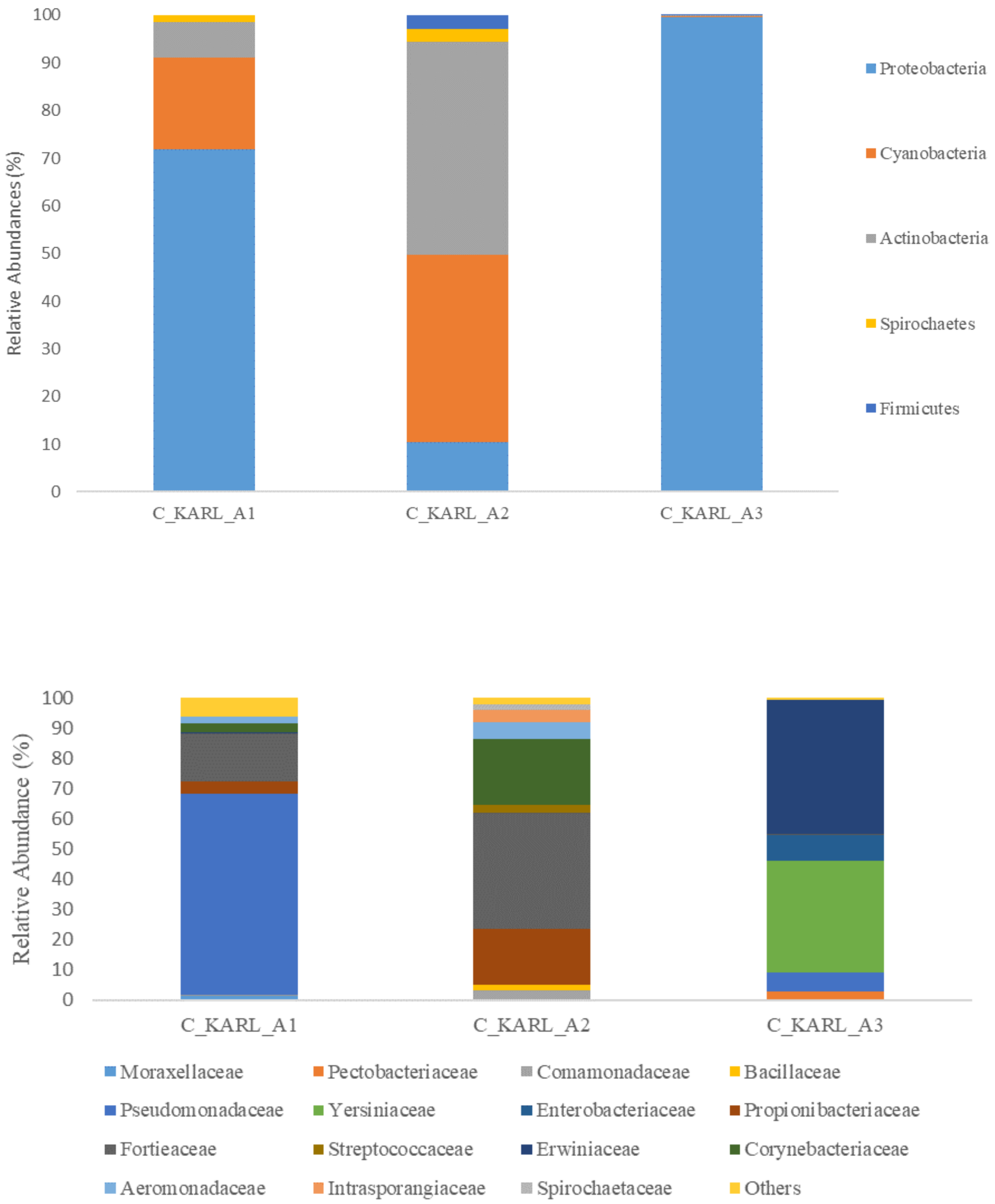

Figure S2. Relative abundance (%) of bacterial phyla (upper) and families (down) of the fish flesh from the three different areas (A1, A2 and A3), as revealed by metabarcoding analysis of 16S rRNA gene.
